# Occupational Exposure to Dust Produced when Milling Thermally Modified Wood

**DOI:** 10.3390/ijerph17051478

**Published:** 2020-02-25

**Authors:** Alena Očkajová, Martin Kučerka, Richard Kminiak, Ľuboš Krišťák, Rastislav Igaz, Roman Réh

**Affiliations:** 1 Faculty of Natural Sciences, Matej Bel University, Banská Bystrica 97401, Slovakia; alena.ockajova@umb.sk (A.O.); martin.kucerka@umb.sk (M.K.); 2 Faculty of Wood Sciences and Technology, Technical University in Zvolen, Zvolen 96001, Slovakia; richard.kminiak@tuzvo.sk (R.K.); igaz@tuzvo.sk (R.I.); reh@tuzvo.sk (R.R.)

**Keywords:** wood dust, occupational exposure, thermowood, oak, spruce, granularity

## Abstract

During production, thermally modified wood is processed using the same machining operations as unmodified wood. Machining wood is always accompanied with the creation of dust particles. The smaller they become, the more hazardous they are. Employees are exposed to a greater health hazard when machining thermally modified wood because a considerable amount of fine dust is produced under the same processing conditions than in the case of unmodified wood. The International Agency for Research on Cancer (IARC) states that wood dust causes cancer of the nasal cavity and paranasal sinuses and of the nasopharynx. Wood dust is also associated with toxic effects, irritation of the eyes, nose and throat, dermatitis, and respiratory system effects which include decreased lung capacity, chronic obstructive pulmonary disease, asthma and allergic reactions. In our research, granular composition of particles resulting from the process of longitudinal milling of heat-treated oak and spruce wood under variable conditions (i.e., the temperature of modification of 160, 180, 200 and 220 °C and feed rate of 6, 10 and 15 m.min^−1^) are presented in the paper. Sieve analysis was used to determine the granular composition of particles. An increase in fine particle fraction when the temperature of modification rises was confirmed by the research. This can be due to the lower strength of thermally modified wood. Moreover, a different effect of the temperature modification on granularity due to the tree species was observed. In the case of oak wood, changes occurred at a temperature of 160 °C and in the case of spruce wood, changes occurred at the temperatures of 200 and 220 °C. At the temperatures of modification of 200 and 220 °C, the dust fraction (i.e., that occurred in the mesh sieves, particles with the size ≤ 0.08 mm) ranged from 2.99% (oak wood, feed rate of 10 m.min^−1^) to 8.07% (spruce wood, feed rate of 6 m.min^−1^). Such particles might have a harmful effect on employee health in wood-processing facilities.

## 1. Introduction

Thermowood is a thermally modified material having interesting properties. High temperature (besides steam and water) is mostly used to modify wood resulting in changes in chemical properties of individual wood components. The result is wood produced with new physical and mechanical properties. Its main positive features include reduced moisture absorption, dimension stability, biological resistance, the attractive appearance of thermally modified wood, the ability to use wood with no surface treatment, a long life cycle (30 years), lower thermal conductivity (suitable for terrace areas), higher surface hardness (abrasive surfaces), greater crack resistance, etc. Thanks to its numerous advantageous properties, thermally modified wood has increasingly been used as a suitable material in the production of floors, stairs, ceilings, paneling, elements of building facades, and humid facilities such as saunas, wellness centres and bathrooms [1,2,3,4,5,6,7,8]. On the other hand, cell wall degradation of hemicelluloses and development of nano- and microcracks are great disadvantages of thermally modified wood that result in reducing the wood strength (especially bending and tensile strength) and toughness limiting the use of thermally modified wood mainly in construction [9,10,11].

During production, thermally modified wood is processed with the same machining operations as unmodified wood. It is necessary to be familiar with the behavior of thermally modified wood in these processes [12,13,14,15]. While there is an increasing amount of performance data for thermally modified tree species, there is limited quantitative information about the machining and processing qualities of thermally modified wood compared to unmodified wood [16]. Thermal modification effects many parameters in the machining process, such as the surface quality of milled wood [17,18], sanded wood [19,20,21], and energy efficiency in cutting/milling/sawing process [22,23] etc.

Wood machining (thermal or native) is always accompanied with creation of dust particles that are more hazardous the smaller they become [24,25]. Particles with the size ≤0.08 mm settle very slowly or do not settle at all (<10µm) and predominate in contaminated air. These particles are dangerous for humans due to their ability to penetrate deep into the airways, including the lung alveoli [26,27,28]. According to some authors, wood dust is more dangerous that formaldehyde [29]. Occupational exposure to wood dust affects 3.6 million workers within the European Union (EU) [30].

Exposure to wood dust is associated with specific health issues. Wood dust is considered carcinogenic to humans (Group 1) according to the International Agency for Research on Cancer (IARC). The IARC states that wood dust causes cancer of the nasal cavity and paranasal sinuses and of the nasopharynx. Wood dust is also associated with toxic effects, irritation of the eyes, nose and throat, dermatitis, and respiratory system effects which include decreased lung capacity, chronic obstructive pulmonary disease, asthma and allergic reactions [31,32,33,34,35,36]. It should be considered that the carcinogenity of dust from thermally modified wood is at least the same as that of dust from unmodified wood, although there are no comprehensive data on the influence of thermal wood modification on the potential harmfulness of dust [37]. However a considerable amount of fine dust are produced under the same processing conditions in the case of machining thermally modified wood when compared to the case of unmodified wood. This result was confirmed in the case of wood sawing [20,38,39] and in the case of wood cutting [40,41]. Also the effect of thermal modification on forming particles or dust must be investigated, either in terms of further utilization or in terms of assessing the health and safety risk [42,43,44,45].

Since problems with wood dust were confirmed by many epidemiologic studies [46,47,48], producers have made great efforts to minimize it. There are three basic ways to reduce the danger of the wood dust occupational exposure, namely use of the proper protective equipment such as face masks, the use of dust control equipment and/or the use of proper technological parameters of processing to minimize the creation of the most dangerous dust (the smallest elements) [49,50,51,52].

The aim of the paper is to determine if there is a statistically significant effect of the temperature of treatment and/or the feed rate on the granular composition of particles from the longitudinal milling of thermally modified spruce and oak wood, and if there are any differences in the composition of particles between thermally modified and unmodified wood.

## 2. Materials and Methods

### 2.1. Samples

Sessile oak (*Quercus petraea*) and Norway spruce (*Picea abies*) were used to produce samples. Sessile oak and Norway spruce were harvested in the area of Vlčí jarok (Budča), 440 m. above sea level (a. s. l.). Radial boards from the trunks of oak and spruce were sawn and subsequently processed to form samples with the dimensions of 20 × 100 mm with the length of approximately 700 mm. The samples were dried to the moisture content of 8%. The process was carried out in the premises of the Research and Development Workshops of the Technical University in Zvolen.

### 2.2. Methods of Thermal Modification and Processing the Samples

Samples with the dimensions of 20 × 100 × 700 mm were thermally modified in the Arboretum in Kostelec nad Černými lesy at the Faculty of Forestry and Wood Sciences (Czech University of Life Science in Prague). The wood samples were modified in a thermal chamber type S400/03 (LAC Ltd., Židlochovice, Czech Republic) using ThermoWood technology. Five pieces of samples were prepared to be used in each mode of thermal modification.

Thermal modification at the individual temperatures, as well as the phases of heating, modifying and cooling of individual tree species and time of application, are described in the paper in more detail [42].

### 2.3. Machines and Equipment

A lower spindle milling machine ZDS-2 (Liptovské strojárne, Slovakia) was used in the experiment. A feeding mechanism Frommia ZMD 252/137 (Maschinenfabrik Ferdinand Fromm, Fellbach, Germany) was used for feeding.

Equipment—milling head FH 45 Staton SZT (Turany, Slovakia), with the parameters: milling machine body with the diameter of 125 mm, diameter with a knife—130 mm, thickness of the component of 45 mm, number of knives—2, material for making the knife—steel Maximum Special 55: 1985/5, rake angle γ = 25°.

### 2.4. Cutting Conditions

cutting speed v_c_ = 40 m·s^−1^

feed rate v_f_ = 6, 10, 15 m·min^−1^

depth of cut = 1 mm.

### 2.5. Granularity Analsis

The method of isokinetic sampling was used to extract samples for granular analysis of particles generated by the process of milling. The samples were extracted from the exhaust system in accordance with the standard STN 9096 (83 4610): “The manual determination of the mass concentration of solid pollutants” during the milling of individual heat-treated wood samples.

The granular composition of particles was determined using the sieve analysis. A standard set of superimposed sieves (2 mm, 1 mm, 0.5 mm, 0.25 mm, 0.125 mm, 0.080 mm, 0.063 mm, 0.032 mm and the bottom) on the vibrating stand of the sieving machine Retsch AS 200c (Retsch GmbH, Haan, Germany) with an adjustable sieving interruption frequency (20 s) and a sieve deflection amplitude (2mm/g), in accordance with STN 153105/STN ISO 3310-1 was used.

The granular composition was determined by weighing the portion that remained in the mesh sieves after sieving with an electronic weighing scale Radwag WPS 510/C/2 (Radwag Balances and Scales, Radom Poland) with a capacity of 510 g and an accuracy of weighing of 0.001 g. Three sieve analyses were conducted in the case of each mode and the results were evaluated statistically. The gathered data were analyzed using the multiple factor analysis of variance (ANOVA) in order to determine the statistical dependence of the percentage of individual granular particles on the monitored parameters.

## 3. Results and Discussion

The results are presented as percentages of particles on individual mesh sieves in the case of both oak wood and spruce wood (Table 1, Table 2 and Table 3).

**Figure 1:**
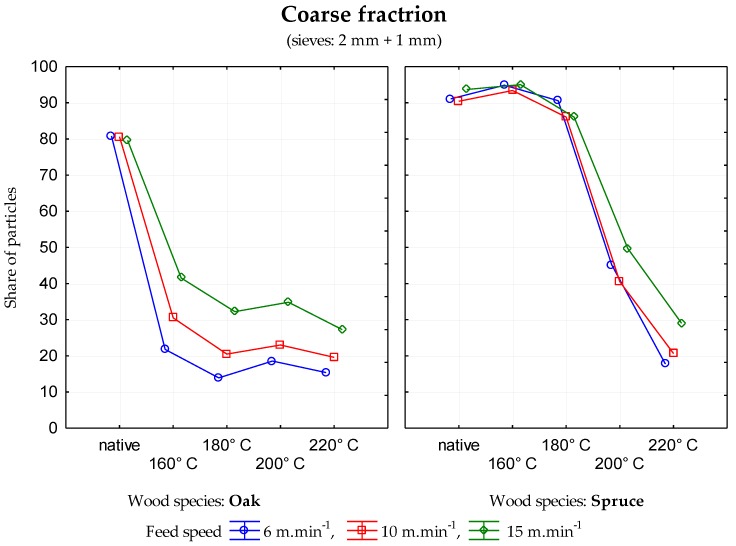
Percentage of particles in the mesh size of 2 mm and 1 mm—coarse fraction depending upon the temperature modification of oak and spruce.

**Figure 2:**
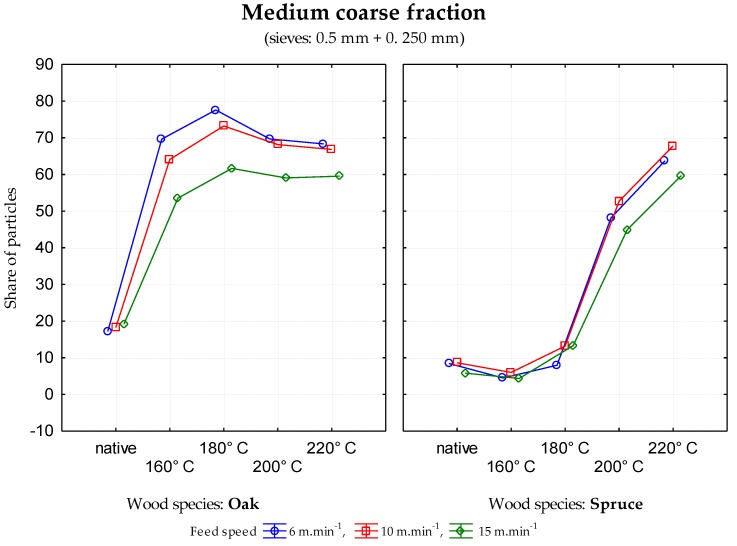
Percentage of particles in the mesh size of 0.5 mm and 0.250 mm—medium coarse fraction depending upon the temperature modification of oak and spruce.

**Figure 3:**
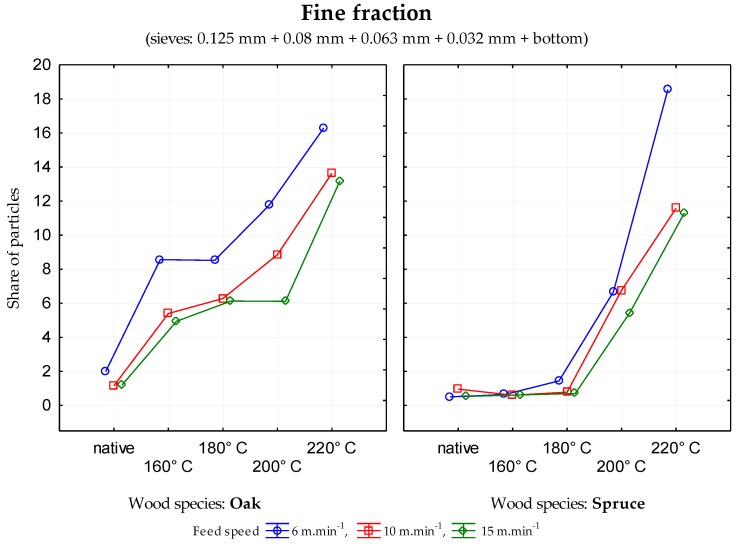
Percentage of particles in the mesh size 0.125 mm, 0.08 mm, 0.063 mm, 0.032 mm and the bottom—fine fraction depending upon temperature the modification of oak and spruce.

**Figure 4:**
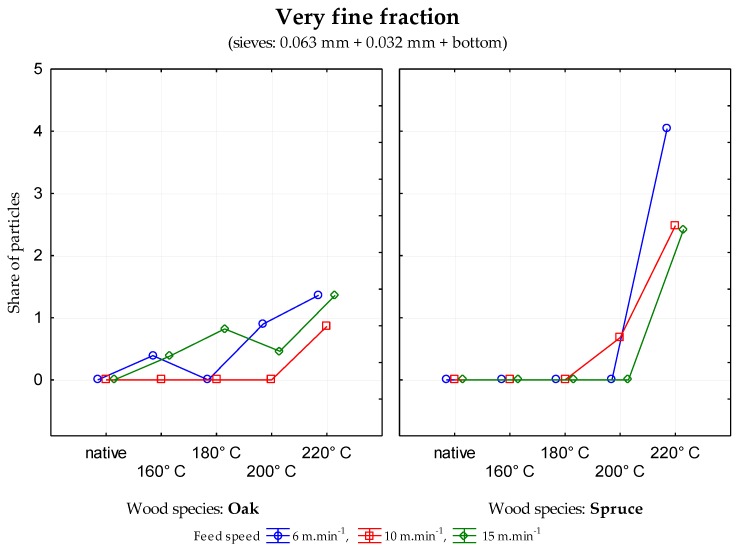
Percentage of very fine particles in the mesh size 0.063 mm, 0.032 mm and the bottom—fine fraction depending upon the modification temperature of oak and spruce.

The data generated indicate that in the case of sessile oak, the percentage of particles in individual mesh sieves was different depending upon the modification temperature. There was a decrease in coarse fraction (Figure 1) and an increase in the medium coarse fraction (Figure 2) and the fine fraction (Figure 3) when the temperature of wood modification increased for all three feed rates. In the case of rising temperature, the percentage of very fine particles and sawdust (Figure 4) increase probably as a result of reducing wood strength and toughness (i.e., an increase in the brittleness of wood). Significant changes in stratification of particles occurred at a temperature of 160 °C.

In the case of Norway spruce, a softwood tree, the pattern of granular composition development of particles differed from those of oak. It is supposed that due to the rising temperature, the changes in chemical components (especially lignin) occurred later compared to broad-leaved trees (with higher percentage of hemicelluloses) that degrade at the lower temperature [53]. Significant changes in stratification of spruce wood particles occurred at a temperature of 200 °C.

In the case of unmodified spruce wood and spruce wood modified at temperatures of 160 and 180 °C, similar percentages of spruce wood particles were found in the mesh sizes of 2 and 1 mm (so called coarse fraction). The individual percentages of particles were not affected by the feed rate in the selected interval. At a temperature modification of 200 °C, the percentage of coarse fraction was approximately halved in comparison to the native wood. At a temperature of 220 °C, the percentage of coarse fraction was approximately only one third in comparison to the native wood.

At the temperatures of modification of 200 and 220 °C, the percentage of particles in the mesh sieves 0.5 and 0.025 (Figure 2) increased significantly in all cases of feed rate. Similar results were observed in the fine fraction (Figure 3).

In terms of safety and health risk, the very fine fraction (Figure 4), (i.e., elements with the size ≤0.08 mm) are considered the most harmful. The generation of very fine particles did not occur during the process of milling the native wood (or their values were very low). In the case of oak wood, it was from 0.23% to 0.40%. In the case of spruce wood and the temperatures of modification of 160 and 180 °C, these particles were not observed either. However when the temperature was further increased, sawdust occurred. In the case of oak and spruce, the highest values 4.3% were at a modification temperature of 220 °C and feed rate of 6 m·min^−1^. In the case of spruce wood, the value was 8.07%. This represents the percent fraction that can result in health and safety risk in the workplaces specialized in woodworking operations [43]. The aforementioned effect is even more significant when using profile knives with higher number of cutting edges.

Statistical analysis was carried out individually for each granular particle size fraction, (i.e., coarse, medium coarse and fine fraction) for data shown in Table 4, Table 5 and Table 6. Following the statistical evaluation, these findings can be stated:

- statistical importance of the factor of tree species was proven;
- statistical importance of the factor of thermal modification was proven;
- the factor tree species is statistically more important than thermal modification;
- statistical importance of the factor of feed rate was not proven.

### 3.1. Tree Species

Results associated with the particle percentage in individual mesh sieves, especially for oak, agree with the results mentioned by [38] for beech wood. The percentage of medium coarse fraction increased in modified beech wood compared to the coarse fraction of beech wood with feed rate.

When comparing the studied tree species, oak and spruce, there were certain differences in the granular composition. This probably is caused by the different contents of main chemical components (cellulose, hemicelluloses and lignin) in softwood and hardwood trees species. Hemicellulose content is higher in softwood species. Hemicelluloses are degraded, to a large extent, at lower temperatures. In the case of oak wood, the content of lignin and cellulose does not change until a temperature modification of 140 °C, a significant change is observed in the content of hemicelluloses with a decrease by approximately 75% [54]. In the case of other hardwood species, e.g., maple wood, this decrease occurs under same conditions, but only 19% in comparison to the native wood [55]. When spruce is thermally modified at the temperatures ranging between 110–270 °C, significant degradation starts at a temperature of 200 °C [53]. Some authors mentioned similar characteristics for degrading the individual wood components due to higher temperature [56,57].

### 3.2. Thermal Modification

A statistically significant effect of thermal modification on granularity of particles resulting from the longitudinal milling of oak and spruce wood was observed. There were increasing percentages of medium coarse, fine and dust fractions when the modification temperature increased. These increases were higher when compared to the coarse fraction of wood not thermally modified. A higher percentage of dust fraction (i.e., particles with the size ≤0.08 mm) representing the health and safety risk can be considered a negative feature of milling heat-treated wood. When milling thermally unmodified wood, these components did not occur or their percentage was too low. These findings are consistent with the statements of the authors dealing with the reduced strength, toughness and increased brittleness of thermally modified wood [1]. Similar results were mentioned by other authors focused on the processes of milling and sawing.

In other research, the particle size distribution obtained in planing (WEINIG Powermat 400) of thermally modified beech wood and steamed beech wood was different. The content of particles (thermally modified wood) smaller than 0.25 mm was up to 7 times higher than in particles of steamed beech wood. These results were the consequences of reduced cutting strength and increased brittleness of thermally modified wood. The percentage of airborne dust particles (particle size <90 μm) was up to 1.26% in chips of thermally modified wood [58].

Similar results were confirmed by Dzurenda [20] when sawing oak wood [59] and beech wood. Thermally modified oak and beech sawdust is finer than unmodified oak and beech sawdust.

By milling thermally modified beech wood and native beech wood using an spindle cutter with STEFF 2034 (altered rpm = 3000, 4500, 6000, 9000; v_c_ = 20, 30, 40 m·s^−1^; v_f_ = 4, 8, 11 m·min^−1^; tool geometry) the cumulative curves are shifted to the left for thermally modified beech wood [14]. Dust fraction, (i.e., particles below 125 μm) was less than 1% in the case of native beech wood samples and less than 4% in thermally modified beech wood samples. When maple wood was thermally modified at a temperature of up to 137.5 ± 2.5 °C, significant changes in granular composition of particles resulting from the process of milling using a CNC (computer numerical control) milling center were not confirmed as it is mentioned by [60]. Similar results of particle granularity using the frame saw when modifying wood with lower temperatures were observed in the case of beech and pine wood [44].

### 3.3. Feed Rate

**Figure 5:**
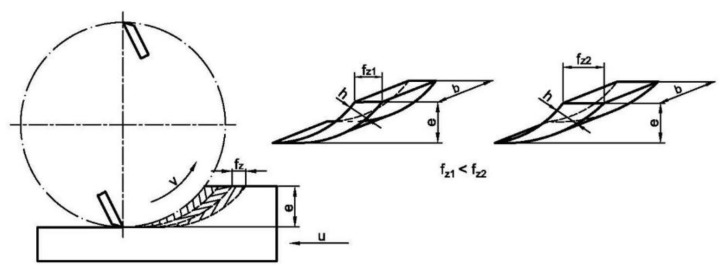
Particles resulting from milling, its different thickness when the feed per tooth is different f_z_. (h—nominal thickness of removed layer, e—depth of cut, b—thickness of sample).

In terms of feed rate and its effect on granularity, the impact of the feed rate itself on the granularity was not statistically significant, even though the higher feed rate corresponded with the greater real thickness of particles. This can be due to the same area size of particles which did not change depending upon the feed rate (f_z1_ < f_z2_, feed per tooth) when the depth of cut “e” remained the same, (Figure 5) or depending upon the shape of mesh sieves used in the analysis. The situation is different at the temperatures above 200 °C, when there is a significant brittleness of wood. It is suspected the increased brittleness is due to the reduction of natural resins and an increase in cell wall cellulose crystallinity [16,61]. Brittleness results from thermal modification (the effect is much more pronounced in coniferous species) and, therefore, there is easier disintegration of wood in the area of minimal chip thickness, which occurs at small values of the sliding speed (6 m·min^−1^). This results in a statistically significant increase in fine and very fine fraction. Therefore, in term of generating fine and very fine particles, also in term of work efficiency, it is strongly recommended to use higher values of feed rate in the case of thermally modified wood milling.

The findings are consistent with other authors who mentioned the ambiguous effect of feed rate on granularity of formed particles in the case of native wood. Feed rate did not have any clear effect on airborne dust emissions in the process of peripheral milling using standard NC (numerical control)-machines [62]. The same conclusions were confirmed by other authors [63]. An increase in feed rate with open cuts (planing, milling) affects a decrease in percentage of particles up to 100 μm in size. However, with a closed cut, the effect of increasing feed rate does not always mean a decrease in smaller particles and it may even have a contrary effect [64].

## 4. Limitations

A milling head with new knives was used in the experiment (because of the desire for reproducibility). The running-in phase of the knives is characterized by the intensive initial wear of the cutting wedge which in terms of the particles formation mechanism may result in minor changes in their granulometric composition. This could have an impact on the removal value from the point of view of the cutting edge recess which may also affect the particle granulometric composition. However, we considered both influences to be less fundamental and, therefore, we did not consider them to be at work.

Due to the installation of the side and face cutter on the shaft of the lower single-spindle milling cutter, a mounting clearance between the clamping hole cutter and the cutter shaft was required (usually 0.5 mm). The mounting clearance caused the offset of the tool placement which can cause small variations in the cut particles’ thickness which in turn affects the particle granulometric composition.

The nature of particles produced in the milling process is foliate. As a result, the particles crumble into smaller pieces within the sieving interval and thus there is a slight shift of the particles granulometric composition downwards.

The maximum feed rate was selected at the level of 15 m.min^−1^. It represents the maximum feed rate to maintain the long-term pace of manual work when loading the workpiece into the machine.

## 5. Conclusions

The following conclusions can be drawn:

1. In the case of longitudinal milling, the percentage of medium coarse, fine and very fine fractions increased as wood modification temperatures increased, especially as compared to the coarse fraction of native wood. Changes in granular composition in the case of oak wood were observed at a temperature of 160 °C and at higher temperatures. In the case of spruce wood, significant changes in granular composition occurred at modification temperatures of 200 and 220 °C.
2. At higher temperatures of modification, a dust fraction (i.e., elements with the size ≤ 0.08 mm) were observed. These particles are dangerous for humans as they are in the air and can penetrate deep into the airways and can get into the lung alveoli. The highest percentage of particles were observed at a temperature of 220 °C.
3. In terms of feed rate, at temperatures above 200 °C a significant brittleness of wood resulting from thermal modification occurred (the effect is much more significant in the case of coniferous trees). This results in a statistically significant increase in fine and very fine fractions with feed rate. Therefore, in terms of reducing fine and very fine particles, it is strongly recommended to use higher values of feed rate in the case of milling thermally modified wood.
4. In the case of the thermally modified wood, even though there is a significant increase of the dust fraction (elements with the size ≤0.08 mm), no particles were recorded of less than 0.032 mm. In terms of implications for practice, for the filtration of an air and dust particle mixture it is necessary to use filtration devices with a capture size limit of 0.032 mm. Cyclone suction is not suitable as the capture size is significantly higher for cyclones.
5. In general, performing a risk assessment and following the hierarchy of control is critical, with elimination of the hazard a priority When working with wood, tools and work processes should be performed that minimize the dust produced. Moreover, tools and the workplace should have appropriate dust extraction and ventilation systems and must be checked and maintained regularly. Monitoring the dust level in the workplace may also be necessary. A well-fitted, effective respirator should also be used if other dust control measures are not practical [65,66,67,68,69,70,71,72]. It is also highly recommended to minimize the exposure time of personnel within the woodworking machine surroundings that create the dangerous dust.

## Figures and Tables

**Table 1:** Percentage of particles on individual mesh sieves in the case of both oak wood and spruce wood with feed rate v_f_ = 6 m·min^−1^.

Sieve	Oak					Spruce				
	Native	160 °C	180 °C	200 °C	220 °C	Native	160 °C	180 °C	200 °C	220 °C
2 mm	59.2	1.17	0.9	1.36	1.81	72.77	83.97	76.81	28.15	3.23
1 mm	21.6	20.62	13	17.19	13.57	18.32	10.9	13.77	17.04	14.52
0.5 mm	11.8	44.75	44.84	39.82	33.94	6.93	3.85	6.52	27.41	34.68
0.25 mm	5.4	24.9	32.74	29.86	34.39	1.49	0.64	1.45	20.74	29.03
0.125 mm	1.6	6.61	7.62	9.05	11.99	0.5	0.64	0.72	5.19	10.48
0.08 mm	0.4	1.56	0.9	1.81	2.94	0	0	0.72	1.48	4.03
0.063 mm	0	0.39	0	0.45	0.68	0	0	0	0	3.23
0.032 mm	0	0	0	0.45	0.68	0	0	0	0	0.81
Bottom	0	0	0	0	0	0	0	0	0	0

**Table 2:** Percentage of particles on individual mesh sieves in the case of both oak wood and spruce wood with feed rate v_f_ = 10 m·min^−1^.

Sieve	Oak					Spruce				
	Native	160 °C	180 °C	200 °C	220 °C	Native	160 °C	180 °C	200 °C	220 °C
2 mm	53.35	3.09	1.67	2.65	3.4	67.94	73.65	58.91	15.54	1.65
1 mm	27.25	27.41	18.83	20.35	16.17	22.49	19.76	27.13	25	19.01
0.5 mm	13.16	43.24	48.12	42.48	35.32	7.18	4.79	10.85	34.46	43.8
0.25 mm	5.08	20.85	25.1	25.66	31.49	1.44	1.2	2.33	18.24	23.97
0.125 mm	0.92	4.63	5.44	7.08	10.64	0.96	0.6	0.78	4.73	6.61
0.08 mm	0.23	0.77	0.84	1.77	2.13	0	0	0	1.35	2.48
0.063 mm	0	0	0	0	0.43	0	0	0	0.68	1.65
0.032 mm	0	0	0	0	0.43	0	0	0	0	0.83
Bottom	0	0	0	0	0	0	0	0	0	0

**Table 3:** Percentage of particles on individual mesh sieves in the case of both oak wood and spruce wood with feed rate v_f_ = 15 m·min^−1^.

Sieve	Oak					Spruce				
	Native	160 °C	180 °C	200 °C	220 °C	Native	160 °C	180 °C	200 °C	220 °C
2 mm	46	7.97	6.12	7.92	6.36	73.16	76.4	51.05	17.01	4.03
1 mm	33.6	33.56	26.12	26.92	20.91	20.53	18.63	34.97	32.65	25
0.5 mm	14.4	36.45	40.82	38.69	32.27	4.21	3.11	11.19	29.93	35.48
0.25 mm	4.8	17.08	20.82	20.36	27.27	1.58	1.24	2.1	14.97	24.19
0.125 mm	0.8	3.8	4.49	4.75	9.55	0.53	0.62	0.7	4.08	6.45
0.08 mm	0.4	0.76	0.82	0.9	2.27	0	0	0	1.36	2.42
0.063 mm	0	0.38	0.41	0.23	0.91	0	0	0	0	1.61
0.032 mm	0	0	0.41	0.23	0.45	0	0	0	0	0.81
Bottom	0	0	0	0	0	0	0	0	0	0

**Table 4:** Multiple factor analysis of variance in the case of coarse fraction.

Coarse Fraction					
Factor	SS	Degrees of Freedom	MS	F-Value	*p*-Value
Feed rate	526.5	2	263.3	0.8141	0.444993
Tree species	11,708.9	1	11,708.9	36.2064	0.000000
Thermal modification	22,382.7	4	5595.7	17.3030	0.000000
Feed rate × Tree species	200.3	2	100.1	0.3096	0.734184
Feed rate × Thermal modification	139.4	8	17.4	0.0539	0.999919
Tree species × Thermal modification	8041.3	4	2010.3	6.2164	0.000118
Feed rate × Tree species × Thermal modification	206.6	8	25.8	0.0798	0.999641
Error	48,509.1	150	323.4		

**Table 5:** Multiple factor analysis of variance in the case of medium coarse fraction.

Medium Coarse Fraction					
Factor	SS	Degrees of Freedom	MS	F-Value	*p*-Value
Feed rate	314.67	2	157.34	3.281	0.040291
Tree species	9544.08	1	9544.08	199.049	0.000000
Thermal modification	14,773.55	4	3693.39	77.028	0.000000
Feed rate × Tree species	152.72	2	76.36	1.593	0.206836
Feed rate × Thermal modification	78.95	8	9.87	0.206	0.989510
Tree species × Thermal modification	6720.21	4	1680.05	35.039	0.000000
Feed rate × Tree species × Thermal modification	159.56	8	19.95	0.416	0.910019
Error	7192.26	150	47.95		

**Table 6:** Multiple factor analysis of variance in the case of fine fraction.

Fine fraction					
Factor	SS	Degrees of Freedom	MS	F-Value	*p*-Value
Feed rate	20.155	2	10.0775	2.1036	0.123295
Tree species	44.086	1	44.0861	9.2028	0.002566
Thermal modification	370.456	4	92.6141	19.3328	0.000000
Feed rate × Tree species	1.301	2	0.6507	0.1358	0.873021
Feed rate × Thermal modification	11.405	8	1.4256	0.2976	0.966621
Tree species × Thermal modification	24.391	4	6.0977	1.2729	0.279897
Feed rate × Tree species × Thermal modification	8.424	8	1.0529	0.2198	0.987335
Error	2012.016	420	4.7905		
